# Bioactive Compounds of Blueberries: Post-Harvest Factors Influencing the Nutritional Value of Products

**DOI:** 10.3390/ijms160818642

**Published:** 2015-08-10

**Authors:** Anna Michalska, Grzegorz Łysiak

**Affiliations:** 1 Institute of Animal Reproduction and Food Research of the Polish Academy of Sciences, Division of Food Science, Str. Tuwima 10, Olsztyn 10-748, Poland; E-Mail: a.michalska@pan.olsztyn.pl; 2 Institute of Agricultural Engineering, Wrocław University of Environmental and Life Sciences, Str. Chelmonskiego 37a, Wroclaw 51-630, Poland; 3 Department of Pomology, Poznan University of Life Sciences, Str. Dąbrowskiego 159, Poznań 60-594, Poland

**Keywords:** antioxidants, drying, maturity, polyphenols processing, quality, storage, *Vaccinium* ssp.

## Abstract

Blueberries, besides having commonly-recognized taste properties, are also a valuable source of health-promoting bioactive compounds. For several decades, blueberries have gained in popularity all over the world, and recent years have seen not only an increase in fresh consumption, but also in the importance of blueberries for the processing industry. Blueberry processing mostly consists of freezing and juicing. Recently, more attention has been drawn to dewatering and drying, which are promising areas for developing novel blueberry products. Processing affects each biologically-active compound in a different way, and it is still unknown what changes those compounds undergo at the molecular level after the application of different processing technologies. This work presents the most recent state of knowledge about the pre-treatment and processing methods applied to blueberries and their influence on the content of biologically-active compounds. The presentation of methods is preceded by a brief overview of the characteristics of the blueberry species, a description of the chemical composition of the fruit and a short note about the main growing areas, production volumes and the management of fruit crops.

## 1. Introduction

Blueberries (*Vaccinium* ssp.) are a species from the family Ericaceae [1], which includes approximately 450 species. Besides cranberries and lingonberries, blueberries were domesticated in the 20th century [2,3]. The popularity of blueberries increased throughout the last decade. In 1990, blueberries were grown only in ten countries [4], whereas in 2011, they were cultivated commercially in 27 countries [5]. The globalization of soft fruit production combined with the development of agricultural mechanization and automation caused a severe problem with the overproduction and unequal distribution of blueberry products throughout the world [6], even making them a political issue. The overproduction of highly perishable soft fruit results in a huge amount of waste. At the same time, it becomes a challenge for the food industry and an objective of food processing technologies to preserve not only the quantity of fruit products, but also to develop novel products and, thus, to offer consumers a broader selection of healthy foodstuffs. Furthermore, in order to promote the consumption of blueberries throughout the year, a number of post-harvest actions are used, including temperature- and atmosphere-control storage and freezing [7,8,9]. A promising area of blueberry processing is temperature-dependent drying performed using conventional [10] and modern technologies, as well as their combination [11], which enhances the processing efficiency and the nutritional quality of blueberry products. The process itself and the conditions applied could be modified in various ways so as to obtain diverse final products with moderate moisture content, thus opening the way for new uses of blueberries in the food industry. In such cases, the quality of new products needs to be thoroughly evaluated, especially when temperature-dependent processes lead to significant changes at a molecular level. In turn, the development of new processing methods and the related findings regarding the changes in the fruit composition constantly necessitate the determination of new fruit quality parameters.

Besides drying, the transformation of blueberries into juices, jellies and powders is still another processing option that ensures the availability of blueberries on the market in a form other than fresh fruit and that may allow one to preserve essential bioactive compounds to a different extent in dependence of the process conditions (*i.e.*, temperature and time). Frequently, in industry, one of the above processing methods is combined with another, so that the knowledge about the changes that occur at the level of bioactive compounds at each single step of the process is essential for the assessment of the final product quality.

Clearly, the application of a broad spectrum of processing technologies and techniques causes alterations in the physical, biological and chemical fruit properties that influence the quality of the blueberry final products. Considering the growing popularity of healthy eating and lifestyles, blueberry processing, regardless of the technology, poses a challenge to scientists and industry, as processing should preserve as many as possible of the biologically-active nutritional components, which are extremely sensitive to any mechanical, physical or chemical treatment [12]. A thorough knowledge of fruit structure and composition is essential for finding an optimum processing method for each blueberry product to be obtained and for defining directions for the further development of fruit processing science. What is more, the knowledge about the influence of processing on the quality of blueberry products may be applied to predict, at the molecular scale, the fate of thermally-labile compounds with documented health-related properties. Taking into account that a growing amount of blueberries undergoes processing, the application of post-harvest handling methods towards maintenance of nutritional quality and food security seem to be important factors for consumers and the industry. Thus, the aim of this review is to present the development of technologies and methods used for blueberry post-harvest processing, especially drying, with the attention focused on the parameters defining the bioactive properties of products. This review focuses on different aspects of blueberry processing, starting from the origin and the content of bioactive compounds and their depletion/accumulation during processing.

## 2. Basic Agronomic Data

### 2.1. Origin

Blueberry growing goes back to the beginning of the 20th century, when Frederick V. Coville selectively bred northern highbush blueberry (*Vaccinium corymbosum* L.) cultivars [13]. In the United States and Canada, also cultivars of other blueberry species are grown: lowbush blueberry (*Vaccinium angustifolium* L.), southern highbush blueberry (*Vaccinium darrowii* Camp.), rabbiteye blueberries (*Vaccinium virgatum* Aiton.), Elliott’s blueberry (*Vaccinium elliottii* Chapm.) and some hybrids between the above or other *Vaccinium* species [2,14]. However, northern highbush blueberries are by far the most frequently-cultivated species, both in the USA and elsewhere in the world, due to high fruit quality and resistance to low temperatures. Apart from the bush structure, the various species differ with respect to soil and climatic requirements, and therefore, hybrids between species grow in importance in breeding programs. For example, *V. angustifolium* can be grown on rocky and dry uplands; *V. virgatum* has high tolerance to drought, high temperatures and a wide range of soil pH levels; *V. elliottii* is very suitable for growing in low chill areas; and *V. virgatum* and *V. darrowii* have no chilling requirements [2,14,15].

New cultivars are obtained through conventional breeding by germplasm selection and hybridization. Currently, no work is being done to release transgenic *Vaccinium* plants for commercial use [16]. However, the transformation protocol for transgenic breeding using *Agrobacterium tumefaciens* strains has been developed since the early 1990s [17]. The first successful transformation of blueberries was carried out by Graham *et al.* in 1996 [18], who used the half-high cultivar “North Country” (*V. corymbosum* L. × *V. angustifolium* Ait.). In 2004, Southern blot confirmed transgenic plants of four commercial varieties of highbush blueberry: “Aurora”, “Bluecrop”, “Brigitta” and “Legacy” [19]. There are many potential types of genes that could be used in the improvement of the *Vaccinium* species [16]. Transgenic breeding of blueberry species could be used to develop various features, such as: resistance*/*tolerance to insects, diseases and herbicides; stress resistance*/*tolerance to drought and cold; and fruit qualities, such as control of ripening, fruit softening, shelf life, nutrition and antioxidants [16,20]. However, the fruit industry is reluctant to introduce transgenic blueberries for commercial release because of expected negative backlash from consumers. In 2001, the North American Blueberry Council along with blueberry sellers around the globe were opposed to any development of transgenic blueberry clones [21].

Nowadays, the main goals for breeders include increasing frost hardiness and reducing chilling requirements, improving tolerance to drought and heat, enhancing the qualities of fruit and increasing the number of parthenocarpic berries [22,23]. Strong stress is made on the breeding of more evenly-ripening cultivars. This feature allows one to better adapt the fruit to machine harvesting for the fresh market [24]. New cultivars are mainly bred in the USA [25,26,27,28]; however, breeding programs are conducted in other countries, as well [29].

### 2.2. Main Growing Regions

According to the FAO [4] and the United States Department of Agriculture (USDA), the United States is the largest blueberry-producing country, with an average production of over 200 thousand tons (2009–2013) accounting for over half of the global production. The second country is Canada (average 93,000 tons), and the third is Poland (10,600 tons). However, the North American Blueberry Council’s (NABC) 2012 report points to a high (and growing) blueberry production in South American countries, mainly Chile [30], a fact that the FAO does not mention. The global production of blueberry is growing rapidly. In 1965, it amounted to only 33 thousand tons, whereas in 2012, it was over 420 thousand tons [4] and, according to NABC, even over 1027 thousand tons [31].

In the USA, blueberry is grown in almost all states, but about 70% of total production comes from Maine, Michigan, New Jersey, Oregon and Georgia [32]. The north-south distribution of production centers allows the prolongation of the harvest period and, consequently, the continuous supply of fresh fruit from the middle of April–October [30]. In Europe, blueberry is grown in almost all EU member countries (68 thousand tons) and in some Eastern European countries (28 thousand tons) [31]. At present, blueberries are produced on all continents.

According to the USA Department of Agriculture (USDA), over 50 blueberry cultivars are currently used for production. They differ in many agronomic features, the most important of them being the harvest date, frost resistance and the required number of chilling hours. Bluecrop, a medium-maturing cultivar with a high yield, is the most popular cultivar in the world (50% of plantings worldwide) [33]. Other popular cultivars include Berkeley, Duke, Elliott, Spartan, Nelson, Herbert and Darrow. Besides their agronomical value, blueberry cultivars are diverse in term of chemical composition and nutritional value. Moreover, the same cultivar can vary in chemical composition and nutritional value depending on where it is grown [34].

### 2.3. Harvest and Management of Fruit Crops

The berry yield per bush is between 8 and 12 kg, which requires up to ten pickings by hand during the harvest season. However, in countries with high labor costs, blueberries are first picked manually 3–5-times, and after a several-day interval, the rest of the fruit is collected by a fruit harvester. Blueberries picked by hand are packaged in plastic transparent boxes and sold for fresh consumption.

After being harvested, regardless of the picking method, blueberries are immediately sorted and placed in cold storage (for either long storage or processing by hydrocooling technology with the addition of sodium hypochlorite solution) at the optimum storage temperature of 0 °C [35] to reduce respiration and fruit dehydration. Most of the fruit is directly intended for fresh consumption. However, due to the growing supply of blueberries and their attractiveness to the processing industry, more and more blueberries are sold in a processed form. In the USA, between 2009 and 2011, about 51 percent of the total blueberry production was sold as fresh fruit, while 48 percent of production was sold as processed fruit [35].

In 2013 in Georgia, one of the USA states leading in blueberry production, more blueberries were processed than sold on the fresh market despite a relatively low crop in that year [36]. Until recently, in Europe, the entire production volume was intended for fresh consumption, but this has changed in the last several years: in 2012, about 8% of blueberry production went for processing, and this was more than the year before [31]. In South America, in 2008, about 20% of total production went for processing, whereas in 2014, when the production doubled, 30% of total crop was processed.

In countries that are large blueberry producers, blueberries not intended for fresh consumption are most often frozen in fluidized tunnel freezers. In the world markets, fresh blueberries are sold in retail packages, and frozen blueberries are sold in bulk packages. The latter, as half-products, are used for processing, *i.e.*, for making jams, conserves or juices [37]. Fruit collected by machine is sorted and stored, and most of it is later sold for industrial processing. The advantage of such a procedure is the effective use of almost the entire crop. Even unripe and defective fruit can be processed. Healthy, but damaged fruit is processed to be used as an ingredient for yogurts or ice creams, whereas unripe fruit is treated as a source of selected biologically-active compounds. The high content of health-promoting substances in fruit is a fact very well known among dietary scientists and consumers, so the interest in fresh fruit and fruit products continues to grow. The latest research has shown that blueberry leaves contain a substance similar to insulin [38,39], which makes blueberry more and more interesting also to the pharmaceutical industry.

## 3. Fruit Structure

**Figure 1:**
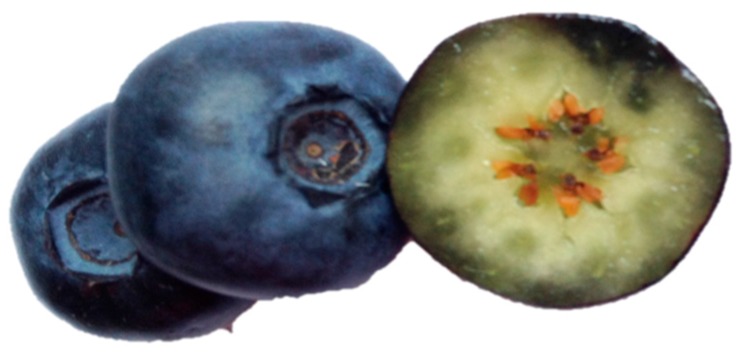
A cross-section of blueberry fruit.

Blueberries, similarly to other soft fruits, have a single layer of epidermis without stomates [40] that is covered by a hydrophobic surface of cuticle and epicuticular wax (Figure 1). This unique part of the fruit plays a valuable role as a protective buffer against external factors (desiccation, infections by pathogenic bacteria and insects, the influence of weather conditions). On the other hand, the waxy outer layer also controls the uptake of water and chemical substances into the fruit, a crucial factor in dewatering/drying processes. Thus, the epicuticular waxy layer not only acts as a significant physiological agent, but also affects the economic aspect of blueberry commodities’ production.

The composition of the blueberry cell wall material is also an important factor that should be considered during the drying of blueberries, regardless of the method. One hundred grams of immature blueberries contain 3.4 and 100 g of ripe blueberries −2.4 g of fresh matter of alcohol-insoluble solids (AIS). The lignins consist of approximately 27% and cellulose of 16% of AIS [41]. What is more, neutral non-cellulosic polysaccharides were found at the level of 0.76 g/100 g fm in immature blueberries and of 0.41 g/100 g fm in ripe fruit [41].

The structure and properties of the outer layer have a strong impact on the water uptake/release from fruit and significantly affect the alteration in the content of biologically-active compounds during processing, as the polyphenols, including anthocyanins, are mainly located directly under the epidermis [42,43]. Depending on the cultivar and the degree of maturity of blueberries, the thickness and composition of the outer layer can vary considerably. Sapers and Phillips [44] reported that a low wax content in the outer layer increases the likelihood of fruit damage during processing (even storage), which leads to a number of leaks and consequently exposes the fruit to the loss of bioactive compounds, particularly valuable anthocyanins [45]. Thus, a thorough selection of blueberry fruit before the processing will allow controlling of the quality of the products obtained, regardless of the further processing method.

## 4. Chemical Composition of Blueberry Fruit

Generally, blueberries in a fresh form consist of water (84%), carbohydrates (9.7%), proteins (0.6%) and fat (0.4%). The average energetic value of a 100-g serving of fresh blueberries is estimated at 192 kJ. Blueberries are also a good source of dietary fiber that constitutes 3%–3.5% of fruit weight. Besides the taste, the main interest in this fruit is due to the moderate vitamin C content, as 100 g of blueberries provide, on average, 10 mg of ascorbic acid, which is equal to 1/3 of the daily recommended intake [46,47].

Previously, numerous scientific reports confirmed that blueberries are an excellent source of health-related compounds, mainly polyphenols [45,48,49,50,51,52]. Several studies confirmed their anti-inflammatory and anti-carcinogenic properties and their cardiovascular protective effects (reviewed by [45]). It is worth mentioning that the antioxidant compounds present in blueberries diminish the risk of coronary diseases, as well as prevent the oxidation of cholesterol, thus lowering the risk of atherosclerosis. These compounds might also avert neurodegenerative disorders [53].

The total content of polyphenols in blueberries ranges from 48 [54] up to 304 mg/100 g of fresh fruit weight (up to 0.3%) [55] and strictly depends on the cultivar [56], growing conditions and maturity [57,58], and its estimation may vary depending on the analytical procedure used [45]. Polyphenols present in blueberries include, *i.e.*, flavonoids, procyanidins (monomeric and oligomeric form) [59], flavonols (*i.e.*, kaempferol, quercetin, myricetin) [56] phenolic acids (mainly hydroxycinnamic acids) and derivatives of stilbenes [60,61]. During the ripening of blueberries, a shift in the pool of total polyphenolic compounds towards anthocyanin synthesis was observed and was in line with a decline in the other individual phenolic components [62], suggesting their important role in terms of the bioactivity of blueberries. In turn, the anthocyanin content has been reported to range from 25 up to 495 mg/100 g of blueberries, and it depends on fruit size, ripening stage, as well as on climatic, pre-harvest environmental conditions and storage [63]. Among berries, the blueberry fruit stands out due to the presence of different types of anthocyanins [64], including malvidin, delphinidin, petunidin, cyanidin and peonidin, with the sugar moieties of glucose, galactose and arabinose. According to some findings, malvidin and delphinidin are the major components and might constitute almost 75% of all identified anthocyanins [65]. However, other findings postulated that the percentage of delphinidin is 27%–40%, malvidin 22%–33%, petunidin 19%–26%, cyanidin 6%–14% and peonidin 1%–5% [42]. The color pigments in blueberries (red, blue, purple) are glycosides of cyaniding, delphinidin and pelargonidin, respectively [66]. Chlorogenic acid present in blueberries is a copigment that enhances the color intensity of anthocyanins [67].

From a practical point of view, the most valuable part of blueberry fruit is its outer layer, as it contains nearly all of the anthocyanins. The polyphenolic compounds are almost exclusively present in the outer layer, but a small content of those compounds was found in flesh and seeds. Their content correlates with the high antioxidant properties of blueberries [43]. Unfortunately, up to now, there has been no report on the changes of particular bioactive components present in different parts of blueberry fruit and their stability during processing.

Regardless of the initial content of the bioactive substances present in blueberries, especially polyphenols, numerous post-harvest factors affect their content and functionality in a diverse way. It was shown that the accumulation of anthocyanins is sustained in overripe berries and might increase after the storage [68], because the loss of firmness of the outer layer enables the water to evaporate faster and at the same time, the application of different processing approaches might reduce/improve their content in the final product.

Generally, there are two key directions of transformation of blueberries into more shelf-stable products. One of them includes juicing, which mostly consists of several preparation steps (*i.e.*, if frozen/thawing, depectinization, pressing, clarification) involving often high-thermal treatments (blanching, pasteurization). In this case, a juice and a press cake are obtained, and from a molecular point of view, this leads to the separation of inherent bioactive compounds present in the fruit in non-equal proportions. Brownmiller *et al.* [69] indicated that 85% of total monomeric anthocyanins were retained after blueberry pressing into juice, whereas further clarification led to the loss of those compounds by almost 25% in comparison to their initial content in raw material. Similarly, other studies confirmed the loss of anthocyanins of even up to 55% during pressing [70]. Thus, if further processing, *i.e.*, juice powdering, is planned, a lower initial content of anthocyanins should be considered, as additional processing might drastically lower the nutritional quality of blueberry products.

The second approach of blueberry conversion into highly shelf-stable products is fruit dehydration. It was reported that commercial blueberry products obtained after thermal processing have similar values of antioxidant activities as fresh blueberries; however, their antiproliferation activities had significantly diminished [71]. Therefore, the properties of the waxy outer layer, its morphology and cellular structure are playing a crucial role in the protection of valuable biologically-active compounds from the influence of negative processing conditions applied during post-harvest handling. Thus, a thorough evaluation of changes in the content of health-related components together with the specification of the applied processes might allow one to design the most efficient method to obtain valuable blueberry products and to create an appropriate strategy for their retention.

## 5. Pre-Treatment of Blueberries

### 5.1. Storage

Storage, often including freezing, is the initial step before the processing of blueberries. According to the literature, fresh blueberries can be successfully stored at 5 °C from two up to seven weeks, depending on the cultivar [8]; however, the recommended optimal temperature is 0 °C [35]. Taking into account that any kind of fruit processing causes alterations in the content of bioactive compounds [72], the initial content of those compounds might be affected directly by the storage conditions (Table 1). Previously, a slightly diminished content of total anthocyanins in line with the increased softness of blueberry fruit stored for two weeks at 5 °C was observed [8], whereas there were no changes in antioxidant capacity measured by the ORAC method. It was shown that an atmosphere composed of 60%–100% O_2_ enhanced the concentration of total phenolics and total anthocyanins in blueberries stored for 35 days at 5 °C [73]. What is more, an increase in total polyphenols, anthocyanins and antioxidant properties during cold storage was noted; however, it was strictly related to the cultivar [74]. Similarly, the storage of blueberries in an atmosphere composed of various CO_2_ and O_2_ concentrations (12:1.5, 12:3, 12:6, 12:12, 18:1.5, 18:3, 18:6, 18:12) increases the anthocyanins content after eight weeks at 0 °C [75]. In the case of blueberry juice, the prolonged six-month storage at 25 °C resulted in a 50% loss in total monomeric anthocyanin content that correlated with the increase of polymeric color [69]. The loss of anthocyanins in blueberry products during 60-day storage was also noted by Srivastava *et al.* [7], and the percentage of their degradation was related to the temperature applied. Indeed, no anthocyanins were detected after storage at 35 °C, whereas significant retention of malvidin (average 34.5%) and peonidin (average 76.7%) for two different blueberry cultivars stored at 23 °C was observed. In the case of ascorbate, there were no changes in the content observed after storage at 0, 10, 20 and 30 °C for up to eight days [76].

Freezing, which might also be used as a pre-treatment method, is another way to ensure a longer availability of blueberries on the market during the year. According to the literature, neither the time (up to six months), nor the temperature (−18 and −35 °C) influenced the total anthocyanin content [65]. This is contrary to Reque *et al.* [77], who found significant losses of anthocyanins in the frozen fruit (59%) after six months of storage at −18 °C. Nevertheless, frozen blueberries were found to have a higher concentration of delphinidin glycosides than fresh ones. In short, the freezing process influenced the extraction of the above-mentioned compounds, as the degradation of the cell structure by ice crystals made their release more efficient [78]. In the industry, the most popular post-harvest technique is an individual quick frozen (IQF) method applied directly after harvesting. A thin layer of fruit is frozen at −40 °C and then packed and kept at this temperature until required [79]. An increase in total polyphenolic compounds in IQF blueberries was indicated as in the case of freezing [71]. It was stated that a slight breakdown of the outer waxy layer after the refrigeration of IQF blueberries might accelerate the rate of drying [80].

### 5.2. Other Pre-Treatment Methods

Blanching is a thermal process designed for inactivating microorganisms and degrading enzymes. The main advantage of this process is the inactivation of polyphenol oxidase (PPO), which reduces the rapid enzymatic browning of fruit while being processed [81], which results in rapid anthocyanin degradation [82]. In the case of blueberries, blanching is also applied to loosen the cellular structure of the outer layer by exposure to hot water or steam for a short period of time [80]. The high-temperature structural changes of the waxy layer, on the one hand, accelerate the water removal from blueberries and, on the other, improve the retention of anthocyanins, thus the antioxidant capacity of blueberries. Brownmiller *et al.* [69] reported that blanching caused no changes in the content of total monomeric anthocyanins in blueberries, whereas antioxidant capacity, as evaluated by the ORAC method, decreased by 26% (Table 1). Other studies presented that stem blanching when applied before further processing into puree/juice/drying affected the anthocyanin content and antioxidant capacity in blueberries due to the release of phenolics concentrated in the outer layer caused by high-temperature-induced tissue disruption [83,84,85]. What is more, the blanching process accelerated the diffusion of anthocyanins from vacuoles present in the outer layer of blueberries into the core of the fruit.

**Table 1:** Changes in blueberry product quality in terms of selected bioactive compounds and quality properties depending on a pre-treatment and the processing method.

**Pre-Treatment Method**		**Influence on the Product Quality**	**Reference**
Storage	Modified atmosphere	Total phenolics (↑)	[74]
		Antioxidant capacity (–)	[8]
		Vitamin C (–)	[76]
Thermal	Freezing	Total phenolics (↑)	[71]
		Total anthocyanins (–)	[69]
		Delphinidin glucoside (↑)	[77]
	Blanching	Total anthocyanins (↑)	[83,84,85]
		Total anthocyanins (–)	[36,69]
		Antioxidant capacity by ORAC (↓)	[36,69]
Mechanical	Cutting (halves/quarters)		
	Scarification		
	Abrasive skin removal	Total phenolics (↓)	[86]
		Vitamin C (↓)	[46]
Chemical	Chemical substances	Organoleptic properties (↓)	[87]
	Natural substances	Total phenolics (–)	[88,89]
		Total anthocyanins (–)	[88,89]
		Antioxidant capacity (–)	[88,89]
**Processing Methods**		**Influence on the Product Quality**	**Reference**
Juicing		Total monomeric anthocyanins (↓)	[69,70]
Dehydration	Osmotic dehydration	Total phenolics (↓)	[8,90]
		Total anthocyanins (↓)	[90]
	Freeze-drying	Vitamins A, C and niacin (↑) *^,^**	[11]
		Polyphenols (ellagic acids, quercetin, naringin, kaempferol) (↑) *^,^**	[11]
		Antioxidant capacity (↑) *^,^**	[11]
	Hot air drying	Total phenolics (↓) ***	[11]
		Total anthocyanins (↓) ***	[11]
		Antioxidant capacity (↓) ***	[11]
	Fluidized bed drying	Total phenolics (↓)	[91]
		Total anthocyanins (↓)	[91]
	Heat pump drying	Total monomeric anthocyanins (↑)	[92]
	Vacuum drying	Volatile compounds (↑) **	[93]
		Total phenolics (↑) **	[93]
		Total anthocyanins (↑) **	[93]
	Radiant zone drying	Total phenolics (–) ***	[94]
		Total anthocyanins (–) ***	[94]
		13 Identified anthocyanins (–) ***	[94]

–, no influence; ↑, increase in the content/properties; ↓, decrease in the content/properties; * compared to microwave vacuum drying; ** compared to hot air drying; *** compared to freeze-drying.

Storage and freezing were found to have a different impact on the bioactive compounds present in blue berries. Regardless of which of these methods is applied before processing, the subsequent handling connected with the dewatering of blueberries to make different ready-to-eat products might greatly affect the content of bioactive compounds in dependence of the conditions applied. Due to the thickness and thus different properties of the waxy layer, numerous mechanical, chemical and thermal pre-treatment methods have been used in order to facilitate the dewatering processes of blueberries [80]. It was confirmed that the basic cellular structure of individual blueberry cultivars differently influences further processing, especially the processing time [86].

To prevent the spoilage of fruit during storage, various methods were used, including non-temperature chemical pre-treatment with naturally-occurring compounds. The latter, *i.e.*, essential oils (p-cymene, linalool, carvacrol, anethole and perillaldehyde) [88] and allyl isothiocyanate were successfully applied to reduce fruit decay [95]. According to the latest mentioned study, the use of the allyl isothiocyanate during 14-day storage did not increase the amounts of phenolic compounds, anthocyanins or the antioxidant capacity of the blueberries; however, it was concluded that the reduction in fruit decay might be due to the allyl isocyanate pro-oxidant action directed against the destruction of microbial cells by free radicals. Although it managed to prevent spoilage during two-week period, no sensory evaluation was conducted. It is worth mentioning that chemical pre-treatment was also applied to improve the content of phenolic compounds. Furthermore, elicitors, chemical compounds that were first used to increase the resistance to pathogens, increase the content of polyphenols [96]. Post-harvest treatment of blueberries with methyl jasmonate (MeJ) resulted in a higher content of total polyphenols in leaves and a higher content of total anthocyanins in fruits [97].

Mechanical pre-treatment of fruit is regarded as a method that changes the fruit’s organoleptic properties to a lesser extent when compared to chemical or thermal preliminary operations. Mechanical pre-treatment was previously applied to berries [87] mainly due to the most important factor related to their overall quality: the acceptability of taste to consumers. The simplest way to prepare fruit before drying is mechanical cutting into halves or quarters. However, in industrial conditions, this solution cannot be successfully applied to blueberries due to their softness. Surface scarification was proposed before drying [86] to form random pin holes, thus increasing the surface porosity that accelerates the drying rate. Other mechanical pre-treatment methods include abrasive skin removal performed in a specially-designed drum [98]. This method is characterized by low energy consumption, so it generates lower capital costs. Additionally, it entails no high-temperature-induced degradation of biologically-active compounds. Importantly, the abrasive preliminary treatment significantly shortens the drying time of blueberries; however, due to the mechanical partial elimination of the outer layer, the loss of phenolic compounds might be expected.

Chemical pre-treatment of blueberries consists of the destruction of the outer layer by softening the waxy surface, which efficiently affects moisture diffusion. It was found that dipping blueberries in sodium peroxide [99], in a mixture of 60% sugar solution and 1% NaCl solution [8], or in alkaline solution of ethyl oleate [100] improved the rate and time of drying and resulted in better moisture removal due to the probable structural collapse and loss of complex arrangements of the waxy layer. The advantages of chemical pre-treatment are the lower costs of processing. Its disadvantage is that the application of chemicals affects the taste of berry products [87] and the final production costs. Osmotic dehydration (OD) of blueberries, as well as other fruits, involves dipping fruit in a concentrated sugar solution for a period of time. The absorption of this solution is strictly connected to its composition and concentration, as well as to the temperature, the time of the process, the structure of the sample and the pressure during treatment [101]. Osmotic dehydration is used for partial non-thermal water removal, which, in the case of blueberries, significantly influences the texture of the final products [102]. Additionally, the fruit quality can be improved by supplementing additional substances to the berry products through the application of concentrated fruit juices [103]. In the case of blueberries, the waxy outer layer influences water permeability, so the thickness of the outer layer, which varies between blueberry cultivars, will impact the effectiveness of the osmotic dehydration of this fruit [104]. It was observed that the concentration of the sucrose solution (47°–70° Brix), the temperature (37–60 °C) and the contact time (0.5–5.5 h) between fruit and sucrose solution affected the moisture loss and solids’ gain after OD processing [105]. With respect to bioactive compounds, a slight loss of anthocyanins was noted [8] due to the soaking and stirring during the OD treatment. Osmotic dehydration of blueberries in a sucrose solution was found to negatively influence the content of anthocyanins and phenolic compounds, as a decrease of approximately 60% was noted [90]. On the other hand, Nikkhah *et al.* [106] observed that sugar concentration up to 20% might protect the stability of anthocyanins, whereas higher sucrose concentrations might cause their degradation. Taking into account that the OD process, if applied before the drying, has a protective effect on the structure of the dried material [107], the thorough evaluation of the OD parameters might minimize the losses of those valuable components.

## 6. Thermal Processing of Blueberries

### 6.1. Temperature-Dependent Dewatering

Although the objectives for the application of drying methods to blueberries are almost the same, heat generation and heat transfer to the berry material are quite different from each other [108]. A comprehensive comparison of the impact of individual processing methods on the stability of bioactive compounds in blueberries is almost impossible due to the application of different methodologies, including pre-treatment methods, equipment and operating conditions, as well as different blueberry varieties and final product quality parameters. The methods already applied to blueberry fruit dehydration are listed in Table 2. However, there is still no precise description of the influence of each single treatment on the nutritional quality of blueberries.

### 6.2. Freeze-Drying

Compared to other common drying methods, freeze-drying is considered one of the gentlest dewatering processes, allowing one to preserve the relatively highest content of biologically-active compounds in fruit [108,109]. Among blueberries processed using four drying methods (freeze-drying, convective air drying, vacuum oven and micro-convection drying), freeze-dried blueberries were characterized by the highest retention of vitamins A and C, niacin and color, a higher rehydration rate and a lower bulk density [79]. Another study revealed that the application of the freeze drying method caused less loss of polyphenols, *i.e.*, ellagic acid, quercetin, phlorizin, naringin and kaempferol, and anthocyanins, and thus resulted in a higher preservation of antioxidant properties than the application of microwave-vacuum and convective drying [11]. Reyes *et al.* [110] proved that the freeze-drying process itself causes the degradation of ascorbic acid in comparison to its initial content in fresh blueberries. The same authors highlighted that different freeze-drying methods had different impacts on the polyphenolic compounds in blueberries, because they observed that the content of those substances decreased after atmospheric freeze-drying, whereas it remained stable after vacuum freeze-drying. With regard to quality attributes, freeze-dried blueberry products received from a sensory panel the best scores for consumer acceptability assessed in terms of appearance, aroma, taste and color [100].

**Table 2:** Recent drying methods applied to blueberry processing.

Drying Method			
Partial drying	Osmotic dehydration		
Single method	Temperature-dependent drying processes	Freeze drying	
		Hot air drying	Fluidized bed drying
			Impingement drying
			Explosion puffing
		Heat pump drying	
		Vacuum drying	
	Other drying technologies	Microwave drying	
		Ohmic heating	
Combined drying methods			

It should be mentioned that this dehydration process of plant material is commonly used for sample preparation, which includes further extraction procedures prior to the identification and quantification of bioactive compounds [111]. Therefore, even in the case of the preparation of blueberry samples in a laboratory, the changes in the chemical composition caused by freeze-drying should be taken into account during the discussion of the results. What is more, the changes in the recovery of the molecules during the extraction process also depend on factors, such as temperature, time, solvents, *etc.* [112]. From an industrial point of view, the main disadvantage of this process is high operational costs arising from the duration of the drying process and affecting the costs of the final products. It should be mentioned that freeze-drying also requires pre-treatment (initial freezing). As a result, the additional costs should be included in fruit preparation before such processing; however, the qualitative edge achieved by the high retention of the bioactive compounds might balance the high cost (and price) of the final product.

### 6.3. Hot Air Drying

Hot air drying is a conventional method that is commonly used for fruit dehydration due to its simplicity. During the process, the plant material is exposed to hot air, and the heat is transferred from the surface to the inside of the sample. The material is usually exposed to relatively high temperatures of drying in the presence of oxygen for a long period of time. The consequence of applying such conditions is the degradation of heat-sensitive compounds, which results in visible shrinkage, noticeable non-enzymatic and enzymatic browning (modification of the final products color), little ability of rehydration and lower nutritional quality [71]. Indeed, at the molecular level, hot air drying of blueberries results in a higher degradation of the total polyphenolic compounds and total anthocyanin content and, as a consequence, in a lower antioxidant capacity when compared to freeze-drying and hot air microwave-vacuum combination drying [11]. It was observed that hot air drying at 70 °C for 10 h caused almost a 60% degradation of anthocyanins in comparison to fresh blueberries, whereas the percentage of the polymerization of those compounds increased by more than 40% [90]. In another study, drying in a cabinet dryer for 5.5 h at 90 °C and reduction during the process to 50 °C resulted in an almost 50% decrease in anthocyanin content in comparison to fresh fruit [8]. Yuan *et al.* [113] proved that drying at 65 °C for 14 days of eight different rabbiteye blueberry cultivars resulted in *ca.* 60% degradation of the total anthocyanins measured by the pH differential method and total phenolics.

One of the hot air drying methods used for blueberry products is fluidized bed drying. During this process, hot air is directed to the drying belt at controlled air velocity, making the product achieve a fluidized state. In a study by Kim *et al.* [102], a fluidized bed dryer set at 170 °C and applied to blueberry drying caused a reduction in the bulk density of dried blueberries when compared to berries dewatered by conventional methods. The application of jet-tube fluidized bed drying resulted in about a 50% degradation of total monomeric anthocyanins after drying at 99 °C and almost an 80% degradation when dried at 116 °C [91].

Impingement drying is another hot air drying method successfully applied to dewater blueberries. In this rapid, simple and efficient method, the hot air or super-heated steam at high velocities is impinged on the surface of fruit [86]. This method was applied to blueberry drying at 85 and 107 °C, and it dried the fruit more quickly than the forced air dryer, but slower than the jet-zone fluidized bed dryer.

Pallas *et al.* [77] applied the continuous explosion puffing system (CEPS) as still another blueberry drying alternative to the hot air drying process employing high temperatures for blueberry dewatering. The optimization of the method parameters was performed; however, no quality parameters connected to the bioactive compounds were evaluated.

Heat pump drying has several advantages over hot air drying, as this method is more energy efficient and provides better quality of food products, because it can be performed at lower temperatures. It is also environmental-friendly due to there being no release of gases into the atmosphere during the processing [114]. This innovative method was successfully applied to dry whole fruit, halves and quarters [92]. It was observed that heat pump drying influenced the content of total monomeric anthocyanins and polymeric color of the extracts obtained, and it was strictly dependent on the shape of blueberries used for drying. 

### 6.4. Vacuum Drying

The benefits of vacuum drying are higher drying rates, a lower drying temperature and an oxygen-deficient processing environment [115,116]. In comparison to conventional air drying, vacuum drying can also result in better maintenance of volatile compounds, which is extremely important for preserving the smell of fresh blueberries in dried products [93]. It was indicated that blueberries dried under vacuum conditions had a greater ability for rehydration and color improvement in comparison to hot air drying [79]. The drying parameters significantly affected the total content of phenolic compounds, anthocyanins and vitamin C, and the optimum process parameters for the retention of those bioactive components were found to be 60 °C at 100 mbar [117]. It was suggested that the drying kinetics and efficiency of vacuum dehydration can be improved by the combination of vacuum drying with microwave power [118].

### 6.5. Microwave Drying

Microwave drying is a method that uses the transfer of electromagnetic waves (*i.e.*, microwaves) through the material to generate heat by the oscillation of molecules. The heat is produced at about the same rate within the entire volume of the materials. Microwave drying was previously applied to blueberries [11,79,107]. Importantly, the application of microwave drying to blueberries resulted in similar sensory properties to those obtained using freeze-drying; however, the time of drying in the microwave method was reduced to half [100]. The advantage of microwave drying is a short drying time in comparison to conventional drying methods; however, the temperature in the material is higher, which may significantly influence the thermolabile bioactive compounds.

### 6.6. Radiant Zone Drying

Radiant zone drying (RZD) was proposed as a novel drying method for blueberry products [94]. The HPLC–DAD analysis of anthocyanins revealed that the application of this method did not change the profile of 13 glycosylated anthocyanins, and the products obtained after RZD were characterized by the same retention of anthocyanins and phenolics as freeze-dried samples.

### 6.7. Infrared Radiation Heating

This dehydration method is more energy efficient, has a greater heat transfer rate and flux that results in a reduced drying time and greater drying rates in comparison to hot air drying [119]. Blueberries IR-heated at 60–80 °C had higher drying rates compared to hot air dried fruit with no visible deterioration of the fruit structure.

### 6.8. Ohmic Heating

In ohmic heating (OH), products are heated by passing alternating electrical current through the sample. The heat is generated by transforming electrical energy into thermal energy, and the process can be performed at rapid rates without the need of a heating medium or surface [120]. In the case of blueberry pulp, the application of this electroconductive heating resulted in the degradation of anthocyanins, namely delphinidin and malvidin, at a level similar to that observed in conventional heating (92 °C) or lower when lower voltage gradients (160–172 V) were used [121]. The authors suggested that, similarly to anthocyanins, a higher voltage used for ohmic heating caused higher degradation of ascorbic acid when compared to conventional drying. This might be explained by the fact that the oxygen generated during the electrolysis of water causes additional oxidation of ascorbate.

### 6.9. Combined Drying Methods

As individual processes have different impacts on blueberries production costs and product quality, combining some of them seems to be more energy efficient and renders products of better quality. A combination of hot air convective drying very commonly used in the post-harvest processing of agricultural products, but involving relatively long drying times [119], and microwave vacuum drying was applied for blueberry pomace drying [122].

## 7. Conclusions

For decades, blueberry breeding programs have been targeted to enhance the commercial traits of blueberries, such as size, color, firmness and productivity [58]. Currently, researchers’ attention has been extended to improving the nutritional aspects and, at the same time, to decreasing the negative consequences of processing conditions that are crucial for the quality of final blueberry products. It was shown that every single step of blueberry processing affects the molecular changes in terms of quantity and quality of biologically-active compounds present in the fruit; however, their exact fate caused by a single process is still unknown. What is more, the probability of the increase/degradation is assumed to be different for each particular biologically-active compound. Currently, along with the growing health-consciousness in society, consumers’ attention is progressively focused on the nutritional quality of foodstuffs, and the need to search for the key factors responsible for the increase/degradation of the bioactive compounds poses a challenge for food chemistry and pharmaceutical design. A thorough analysis at the molecular level of biologically-active blueberry components might greatly contribute to their application in both industries and, thus, provide a path for developing novel technologies.

## References

[1] Luby J.J., Ballington J.R., Draper A.D., Pliska K., Austin M.E., Moore J.N., Ballington J.R. (1999). Blueberries and cranberries (*Vaccinium*). Genetic Resources of Temperate Fruit and Nut Crops.

[2] Westwood M.N. (1993). Temperate-Zone Pomology. Physiology and Culture.

[3] Lyrene P.M., Vorsa N., Ballington J.R. (2003). Polyploidy and sexual polyploidization in the genus *Vaccinium*. Euphytica.

[4] Statistics Division, Food and Agriculture Organization of the United Nations Production. http://faostat3.fao.org/home/E.

[5] Evans E.A., Ballen F.H. An Overview of US Blueberry Production, Trade, and Consumption, with Special Reference to Florida. 2014. UF/IFAS Extention. http://edis.ifas.ufl.edu/fe952.

[6] Institution of Mechanical Engineers Global food. Waste not, Want not. http://www.imeche.org/docs/default-source/reports/Global_Food_Report.pdf?sfvrsn=0.

[7] Srivastava A., Akoh C.C., Yi W., Fischer J., Krewer G. (2007). Effect of storage conditions on the biological activity of phenolic compounds of blueberry extract packed in glass bottles. J. Agric. Food Chem..

[8] Lohachoompol V., Srzednicki G., Craske J. (2004). The change of total anthocyanins in blueberries and their antioxidant effect after drying and freezing. J. Biomed. Biotechnol..

[9] Forney C.F. Postharvest issues in blueberry and cranberry and methods to improve market-life. Proceedings of the ISHS Acta Horticulturae 810: IX International Vaccinium Symposium.

[10] Sablani S.S., Andrews P.K., Davies N.M., Walters T., Saez H., Bastarrachea L. (2011). Effects of air and freeze drying on phytochemical content of conventional and organic berries. Dry Technol..

[11] Mejia-Meza E., Yanez J.A., Davies N.M., Rasco B., Younce F., Remsberg C., Clary C. (2008). Improving nutritional value of dried blueberries (*Vaccinium corymbosum* L.) combining microwave-vacuum, hot-air drying and freeze drying technologies. Int. J. Food Eng..

[12] Rothwell J.A., Medina-Remón A., Pérez-Jiménez J., Neveu V., Knaze V., Slimani N., Scalbert A. (2015). Effects of food processing on polyphenol contents: A systematic analysis using Phenol-Explorer data. Mol. Nutr. Food Res..

[13] Mainland C.M., Frederick V. (2012). Coville and the history of North American highbush blueberry culture. Int. J. Fruit Sci..

[14] Prodorutti D., Pertot I., Giongo L., Gessler C. (2007). Highbush blueberry: Cultivation, protection, breeding and biotechnology. Eur. J. Plant Sci. Biotechnol..

[15] Ehlenfeldt M.K., Ogden E.L., Rowland L.J., Vinyard B. (2006). Evaluation of midwinter cold hardiness among 25 rabbiteye blueberry cultivars. HortScience.

[16] Song G.Q., Hancock J.F. (2012). Recent advances in blueberry transformation. Int. J. Fruit Sci..

[17] Rowland L.J. (1990). Susceptibility of blueberry to infection by *Agrobacterium tumefaciens*. HortScience.

[18] Graham J., Greig K., McNicol R.J. (1996). Transformation of blueberry without antibiotic selection. Ann. Appl. Biol..

[19] Song G.-Q., Sink K.C. (2004). Agrobacterium tumefaciens-mediated transformation of blueberry (*Vaccinium corymbosum* L.). Plant Cell Rep..

[20] Giovannoni J.J. (2004). Genetic regulation of fruit development and ripening. Plant Cell.

[21] North American Blueberry Council NABC position on transgenic plants or GMO’s. http://www.smallfruits.org/blueberries/production/NABCposition.htm.

[22] Ehlenfeldt M.K. (2012). Breeding for parthenocarpic fruit development in blueberry. Int. J. Fruit Sci..

[23] NeSmith D.S. Blueberry Cultivar Development at the University of Georgia—A Progress Report for 2012. http://smallfruits.org/Blueberries/production/alap12report.pdf.

[24] Olmstead J.W., Finn C.E. (2014). Breeding highbush blueberry cultivars adapted to machine harvest for the fresh market. HortTechnology.

[25] NeSmith D.S. Performance of old and new rabbiteye blueberry cultivars from the University of Georgia breeding program. Proceedings of the ISHS Acta Horticulturae 715: VIII International Symposium on Vaccinium Culture.

[26] Ballington J.R., Rooks S.D., Cline W.O., Meyer J.R., Milholand R.D. The North Carolina State University blueberry breeding program—Toward V. X covilleanum?. Proceedings of the ISHS Acta Horticulturae 446: VI International Symposium on Vaccinium Culture.

[27] Lyrene P., Janick J. (2007). Breeding southern highbush blueberries. Plant Breeding Reviews.

[28] Brevis P.A., Bassil N.V., Ballington J.R., Hancock J.F. (2008). Impact of wide hybridization on highbush blueberry breeding. J. Am. Soc. Hortic. Sci..

[29] Scalzo J., Stevenson D., Hedderley D. (2013). Blueberry estimated harvest from seven new cultivars: fruit and anthocyanins. Food Chem..

[30] US Highbush Blueberry Council Where blueberries grow. http://www.blueberrycouncil.org/blueberry-facts/where-blueberries-grow/.

[31] Brazelton C. World Blueberry Acreage and Production. http://www.chilealimentos.com/2013/phocadownload/Aprocesados_congelados/nabc_2012-world-blueberry-acreage-production.pdf.

[32] United States Department of Agriculture Economic Research Service Fruit and tree nuts yearbook tables: Berries: Blueberries. http://www.ers.usda.gov/data-products/fruit-and-tree-nut-data/yearbook-tables.aspx#40875.

[33] Moore J.N. (1993). Blueberry cultivars of North America. HortTechnology.

[34] Connor A.M., Luby J.J., Tong C.B.S., Finn C.E., Hancock J.F. (2002). Genotypic and environmental variation in antioxidant activity, total phenolic content, and anthocyanin content among blueberry cultivars. J. Am. Soc. Hortic. Sci..

[35] Crisosto C.H., Mitcham E.J., Kader A.A. (1998). Perishables Handling #95.

[36] United States Department of Agriculture Economic Research Service Yearbook Tables 2014. http://www.ers.usda.gov/datafiles/FruitTreeNuts_YearbookTables/Berries/Table-D2.xlsx.

[37] Villata M. (1998). Cultivated blueberries-a true-blue baking ingredient. Cereal Food World.

[38] Martineau L.C., Couture A., Spoor D., Benhaddou-Andaloussi A., Harris C., Meddah B., Leduc C., Burt A., Vuong T., Mai Le P. (2006). Anti-diabetic properties of the Canadian lowbush blueberry *Vaccinium angustifolium* Ait. Phytomedicine.

[39] Wang L.J., Wu J., Wang H.X., Li S.S., Zheng X.C., Du H., Xu Y.J., Wang L.S. (2015). Composition of phenolic compounds and antioxidant activity in the leaves of blueberry cultivars. J. Funct. Foods.

[40] Giongo L., Poncetta P., Loretti P., Costa F. (2013). Texture profiling of blueberries (*Vaccinium* spp.) during fruit development, ripening and storage. Postharvest Biol. Technol..

[41] Hamauzu Y., Mizuno Y. (2011). Non-extractable procyanidins and lignin are important factors in the bile acid binding and radical scavenging properties of cell wall material in some fruits. Plant Foods Hum. Nutr..

[42] Cho M.J., Howard L.R., Prior R.L., Clark J.R. (2004). Flavonoid glycosides and antioxidant capacity of various blackberry, blueberry and red grape genotypes determined by high-performance liquid chromatography/mass spectrometry. J. Sci. Food Agric..

[43] Lee J., Wrolstad R.E. (2004). Extraction of anthocyanins and polyphenolics from blueberry processing waste. J. Food Sci..

[44] Sapers G.M., Phillips J.G. (1985). Leakage of anthocyanins from skin of raw and cooked highbush blueberries (*Vaccinium*
*corymbosum* L.). J. Food Sci..

[45] Routray W., Orsat V. (2011). Blueberries and their anthocyanins: Factors affecting biosynthesis and properties. Compr. Rev. Food Sci. Food Saf..

[46] Prior R.L., Cao G., Martin A., Sofic E., McEwen J., O’Brien C., Lischner N., Ehlenfeldt M., Kalt W., Krewer G. (1998). Antioxidant capacity as influenced by total phenolic and anthocyanin content, maturity, and variety of *vaccinium* species. J. Agric. Food Chem..

[47] National Health and Medical Research Council (NHMRC) Nutrient relevance values for Australia and New Zealand: Including recommended dietary intakes. https://www.nhmrc.gov.au/_files_nhmrc/publications/attachments/n35.pdf.

[48] Zafra-Stone S., Yasmin T., Bagchi M., Chatterjee A., Vinson J.A., Bagchi D. (2007). Berry anthocyanins as novel antioxidants in human health and disease prevention. Mol. Nutr. Food Res..

[49] Norberto S., Silva S., Meireles M., Faria A., Pintado M., Calhau C. (2013). Blueberry anthocyanins in health promotion: A metabolic overview. J. Funct. Foods.

[50] Correa-Betanzo J., Allen-Vercoe E., McDonald J., Schroeter K., Corredig M., Paliyath G. (2014). Stability and biological activity of wild blueberry (*Vaccinium angustifolium*) polyphenols during simulated *in vitro* gastrointestinal digestion. Food Chem..

[51] Giacalone M., di Sacco F., Traupe I., Pagnucci N., Forfori F., Giunta F., Preedy R.R.W.R. (2015). Blueberry polyphenols and neuroprotection. Bioactive Nutraceuticals and Dietary Supplements in Neurological and Brain Disease.

[52] Diaconeasa Z., Leopold L., Rugină D., Ayvaz H., Socaciu C. (2015). Antiproliferative and antioxidant properties of anthocyanin rich extracts from blueberry and blackcurrant juice. Int. J. Mol. Sci..

[53] Ramassamy C. (2006). Emerging role of polyphenolic compounds in the treatment of neurodegenerative diseases: A review of their intracellular targets. Eur. J. Pharmacol..

[54] Ehlenfeldt M.K., Prior R.L. (2001). Oxygen radical absorbance capacity (ORAC) and phenolic and anthocyanin concentrations in fruit and leaf tissues of highbush blueberry. J. Agric. Food Chem..

[55] Moyer R.A., Hummer K.E., Finn C.E., Frei B., Wrolstad R.E. (2002). Anthocyanins, phenolics, and antioxidant capacity in diverse small fruits: Vaccinium, rubus, and ribes. J. Agric. Food Chem..

[56] Taruscio T.G., Barney D.L., Exon J. (2004). Content and profile of flavanoid and phenolic acid compounds in conjunction with the antioxidant capacity for a variety of northwest *Vaccinium* berries. J. Agric. Food Chem..

[57] Zadernowski R., Naczk M., Nesterowicz J. (2005). Phenolic acid profiles in some small berries. J. Agric. Food Chem..

[58] Castrejón A.D.R., Eichholz I., Rohn S., Kroh L.W., Huyskens-Keil S. (2008). Phenolic profile and antioxidant activity of highbush blueberry (*Vaccinium corymbosum* L.) during fruit maturation and ripening. Food Chem..

[59] Gu L., Kelm M., Hammerstone J.F., Beecher G., Cunningham D., Vannozzi S., Prior R.L. (2002). Fractionation of polymeric procyanidins from lowbush blueberry and quantification of procyanidins in selected foods with an optimized normal-phase HPLC–MS fluorescent detection method. J. Agric. Food Chem..

[60] Wu X., Kang J., Tuberoso C. (2012). Blueberries: Major phytochemicals and potential health effects in cardiovascular diseases. Berries: Properties, Consumption and Nutrition.

[61] Howard L., Hagar T., Zhao Y. (2007). Berry fruit phytochemicals. Berry Fruit: Value Added Products for Health Promotion.

[62] Kalt W., Lawand C., Ryan D.A.J., McDonald J.E., Donner H., Forney C.F. (2003). Oxygen radical absorbing capacity, anthocyanin and phenolic content of highbush blueberries (*Vaccinium corymbosum* L.) during ripening and storage. J. Am. Soc. Hortic. Sci..

[63] Mazza G., Miniati E. (1993). Anthocyanins in Fruits, Vegetables and Grains.

[64] Gao L., Mazza G. (1994). Quantitation and distribution of simple and acylated anthocyanins and other phenolics in blueberries. J. Food Sci..

[65] Scibisz I., Mitek M. (2007). Influence of freezing process and frozen storage on anthocyanin contents of highbush blueberries. Food Sci. Technol. Qual..

[66] Lee J., Durst R.W., Wrolstad R.E. (2005). Determination of total monomeric anthocyanin pigment content of fruit juices, beverages, natural colorants, and wines by the pH differential method: collaborative study. J. AOAC Int..

[67] Mazza G., Brouillard R. (1990). The mechanism of co-pigmentation of anthocyanins in aqueous solutions. Phytochemistry.

[68] Kalt W., Howell A., Duy J.C., Forney C.F., McDonald J.E. (2001). Horticultural factors affecting antioxidant capacity of blueberries and other small fruit. HortTechnology.

[69] Brownmiller C., Howard L.R., Prior R.L. (2008). Processing and storage effects on monomeric anthocyanins, percent polymeric color, and antioxidant capacity of processed blueberry products. J. Food Sci..

[70] Lee J., Durst R.W., Wrolstad R.E. (2002). Impact of juice processing on blueberry anthocyanins and polyphenolics: Comparison of two pretreatments. J. Food Sci..

[71] Schmidt B.M., Erdman J.W., Lila M.A. (2005). Effects of food processing on blueberry antiproliferation and antioxidant activity. J. Food Sci..

[72] Nicoli M.C., Anese M., Parpinel M. (1999). Influence of processing on the antioxidant properties of fruit and vegetables. Trends Food Sci. Technol..

[73] Zheng Y., Wang C.Y., Wang S.Y., Zheng W. (2003). Effect of high-oxygen atmospheres on blueberry phenolics, anthocyanins, and antioxidant capacity. J. Agric. Food Chem..

[74] Connor A.M., Luby J.J., Hancock J.F., Berkheimer S., Hanson E.J. (2002). Changes in fruit antioxidant activity among blueberry cultivars during cold-temperature storage. J. Agric. Food Chem..

[75] Krupa T., Tomala K. (2006). Effects of storage condition on anthocyanin content and antioxidative activity in highbush blueberries fruit. Food Sci. Technol. Qual..

[76] Kalt W., Forney C.F., Martin A., Prior R.L. (1999). Antioxidant capacity, vitamin C, phenolics, and anthocyanins after fresh storage of small fruits. J. Agric. Food Chem..

[77] Reque P.M., Steffens R.S., Jablonski A., Flôres S.H., de Rios A.O., de Jong E.V. (2014). Cold storage of blueberry (*Vaccinium* spp.) fruits and juice: Anthocyanin stability and antioxidant activity. J. Food Compos. Anal..

[78] Stewart K., Somogyi L., Barrett D.M., Hui Y.H. (1996). Processing in cranberry, blueberry, currant, and gooseberry. Processing Fruits.

[79] Yang C.S.T., Atallah W.A. (1985). Effect of four drying methods on the quality of intermediate moisture lowbush blueberries. J. Food Sci..

[80] George S.D., Cenkowski S., Muir W.E. A review of drying technologies for the preservation of nutritional compounds in waxy skinned fruit. Proceedings of the 2004 North Central ASAE/CSAE Conference.

[81] Tomás-Barberán F.A., Espín J.C. (2001). Phenolic compounds and related enzymes as determinants of quality in fruits and vegetables. J. Sci. Food Agric..

[82] Kader F., Rovel B., Girardin M., Metche M. (1997). Mechanism of browning in fresh highbush blueberry fruit (*Vaccinium corymbosum* L.) role of blueberry polyphenol oxidase, chlorogenic acid and anthocyanins. J. Sci. Food Agric..

[83] Sablani S.S., Andrews P.K., Davies N.M., Walters T., Saez H., Syamaladevi R.M., Mohekar P.R. (2010). Effect of thermal treatments on phytochemicals in conventionally and organically grown berries. J. Sci. Food Agric..

[84] Brambilla A., Maffi D., Rizzolo A. Study of the influence of berry blanching on syneresis in blueberry purées. Procedia Food Science, Proceedings of the 11th International Congress on Engineering and Food (ICEF11).

[85] Rossi M., Giussani E., Morelli R., Lo Scalzo R., Nani R.C., Torreggiani D. (2003). Effect of fruit blanching on phenolics and radical scavenging activity of highbush blueberry juice. Food Res. Int..

[86] Yemmireddy V.K., Chinnan M.S., Kerr W.L., Hung Y.C. (2013). Effect of drying method on drying time and physico-chemical properties of dried rabbiteye blueberries. LWT Food Sci. Technol..

[87] Grabowski S., Marcotte M., Welti-Chanes W., Velez-Ruiz F., Barbosa-Canovas G.V. (2003). Pre-treatment efficiency in osmotic dehydration of cranberries. Transport Phenomena in Food Processing.

[88] Wang C.Y., Wang S.Y., Chen C. (2008). Increasing antioxidant activity and reducing decay of blueberries by essential oils. J. Agric. Food Chem..

[89] Wang S.Y., Chen C.T., Yin J.J. (2010). Effect of allyl isothiocyanate on antioxidants and fruit decay of blueberries. Food Chem..

[90] Stojanovic J., Silva J.L. (2007). Influence of osmotic concentration, continuous high frequency ultrasound and dehydration on antioxidants, colour and chemical properties of rabbiteye blueberries. Food Chem..

[91] Pallas L.A., Pegg R.B., Shewfelt R.L., Kerr W.L. (2012). The role of processing conditions on the color and antioxidant retention of jet tube fluidized bed–dried blueberries. Dry. Technol..

[92] Horszwald A., Alves-Filho O. Innovative blueberry heat pump drying technology: Evaluation of anthocyanins, antioxidant capacity and kinetics. Proceedings of the 6th Nordic Drying Conference.

[93] Sagar V.R., Suresh Kumar P. (2010). Recent advances in drying and dehydration of fruits and vegetables: A review. J. Food Sci. Technol..

[94] Chakraborty M., Savarese M., Harbertson E., Harbertson J., Ringer K.L. (2010). Effect of the novel radiant zone drying method on anthocyanins and phenolics of three blueberry liquids. J. Agric. Food Chem..

[95] Wang S.Y., Chen C.T. (2010). Effect of allyl isothiocyanate on antioxidant enzyme activities, flavonoids and post-harvest fruit quality of blueberries (*Vaccinium corymbosum* L., cv. Duke). Food Chem..

[96] Ruiz-García Y., Gómez-Plaza E. (2013). Elicitors: A tool for improving fruit phenolic content. Agriculture.

[97] Percival D., MacKenzie J.L. (2007). Use of plant growth regulators to increase polyphenolic compounds in the wild blueberry. Can. J. Plant Sci..

[98] Somsong P., Srzednicki G., Konczak I., Lohachoompol V. Effects of preconditioning on quality of dried blueberries. Proceedings of the 10th International Working Conference on Stored Product Protection.

[99] Shi J., Pan Z., McHugh T.H., Wood D., Zhu Y., Avena-Bustillos R.J., Hirschberg E. (2008). Effect of berry size and sodium hydroxide pretreatment on the drying characteristics of blueberries under infrared radiation heating. J. Food Sci..

[100] Venkatachalapathy K., Raghavan S.G.V. (1998). Microwave drying of osmotically dehydrated blueberries. J. Microw. Power EE.

[101] Shi X.Q., Fito P., Chiralt A. (1995). Influence of vacuum treatment on mass transfer during osmotic dehydration of fruits. Food Res. Int..

[102] Kim M.H., Toledo R.T. (1987). Effect of osmotic dehydration and high temperature fluidized bed drying on properties of dehydrated rabbiteye blueberries. J. Food Sci..

[103] Calín-Sánchez Á., Kharaghani A., Lech K., Figiel A., Carbonell-Barrachina Á.A., Tsotsas E. (2014). Drying inetics and microstructural and sensory properties of black chokeberry (*Aronia melanocarpa*) as affected by drying method. Food Bioprocess. Technol..

[104] Schönherr J. (1976). Water permeability of isolated cuticular membranes: The effect of cuticular waxes on diffusion of water. Planta.

[105] Nsonzi F., Ramaswamy H.S. (1998). Osmotic dehydration kinetics of blueberries. Dry. Technol..

[106] Nikkhah E., Khayamy M., Heidari R., Jamee R. (2007). Effect of sugar treatment on stability of anthocyanin pigments in berries. J. Biol. Sci..

[107] Feng H., Tang J., Mattinson D.S., Fellman J.K. (1999). Microwave and spouted bed drying of frozen blueberries: The effect of drying and pretreatment methods on physical properties and retention of flavor volatiles. J. Food Process. Preserv..

[108] Ratti C. (2001). Hot air and freeze-drying of high-value foods: A review. J. Food Eng..

[109] Irzyniec Z., Klimczak J., Michalowski S. (1995). Freeze-drying of the black currant juice. Dry. Technol..

[110] Reyes A., Evseev A., Mahn A., Bubnovich V., Bustos R., Scheuermann E. (2011). Effect of operating conditions in freeze-drying on the nutritional properties of blueberries. Int. J. Food Sci. Nutr..

[111] Prior R.L., Lazarus S.A., Cao G., Muccitelli H., Hammerstone J.F. (2001). Identification of procyanidins and anthocyanins in blueberries and cranberries (*Vaccinium* spp.) using high-performance liquid chromatography/mass spectrometry. J. Agric. Food Chem..

[112] Spigno G., Tramelli L., de Faveri D.M. (2007). Effects of extraction time, temperature and solvent on concentration and antioxidant activity of grape marc phenolics. J. Food Eng..

[113] Yuan W., Zhou L., Deng G., Wang P., Creech D., Li S. (2011). Anthocyanins, phenolics and antioxidant capacity of *Vaccinium* L. in Texas, USA. Pharm. Crops.

[114] Perera C.O., Rahman M.S. (1997). Heat pump dehumidifier drying of food. Trends Food Sci. Technol..

[115] Rahman M.S., Al-Shamsi Q.H., Bengtsson G.B., Sablani S.S., Al-Alawi A. (2009). Drying kinetics and allicin potential in garlic slices during different methods of drying. Dry. Technol..

[116] Wu L., Orikasa T., Ogawa Y., Tagawa A. (2007). Vacuum drying characteristics of eggplants. J. Food Eng..

[117] Sumic Z., Tepic A., Jokic S., Malbasa R. (2015). Optimization of frozen wild blueberry vacuum drying process. Hem. Ind..

[118] Jiang H., Zhang M., Bhandari B., Bansal N., Zhang M., Schuck P. (2013). Fruit and vegetable powders. Handbook of Food Powders.

[119] Shi J., Pan Z., McHugh T.H., Wood D., Hirschberg E., Olson D. (2008). Drying and quality characteristics of fresh and sugar-infused blueberries dried with infrared radiation heating. LWT Food Sci. Technol..

[120] Sastry S.K., Barach J.T. (2000). Ohmic and inductive heating. J. Food Sci..

[121] Sarkis J., Jaeschke D., Tessaro I., Marczak L.D.F. (2013). Effects of ohmic and conventional heating on anthocyanin degradation during the processing of blueberry pulp. LWT Food Sci. Technol..

[122] Zielinska M., Horszwald A. A multi-stage convective and microwave vacuum drying of blueberry fruits and pomace: Color, total anthocyanins, total phenolics and antioxidant capacity. Proceedings of the 1st International Conference on Food Properties (ICFP2014).

